# Relapsing intracranial plasma cell granuloma: A case report

**DOI:** 10.3892/ol.2013.1722

**Published:** 2013-12-02

**Authors:** JACLYN J. RENFROW, JERRY W. MITCHELL, MICHAEL GOODMAN, LEIGH A. MELLEN, JOHN A. WILSON, RYAN T. MOTT, GLENN J. LESSER

**Affiliations:** 1 Wake Forest University School of Medicine, Winston-Salem, NC 27157, USA; 2 The Mark H. Zangmeister Center, Columbus, OH 43219, USA; 3 Department of Internal Medicine, Section on Hematology and Oncology, Comprehensive Cancer Center, Wake Forest University School of Medicine, Winston-Salem, NC 27157, USA; 4 Hattiesburg Clinic, Department of Pathology, Hattiesburg, MS 39401, USA; 5 Department of Neurosurgery, Wake Forest University School of Medicine, Winston-Salem, NC 27157, USA; 6 Department of Pathology, Wake Forest University School of Medicine, Winston-Salem, NC 27157, USA

**Keywords:** plasma cell granuloma, inflammatory pseudotumor, glucocorticoids, erythrocyte sedimentation rate, primary intracranial plasma cell granuloma

## Abstract

Plasma cell granuloma is a pathological entity reported in nearly every organ system; however, intracranial cases remain rare. In the current case report, we present a case of intracranial plasma cell granuloma with the longest known follow-up period in the literature. Medical follow-up over 14 years, detailing four recurrences following the patient’s initial presentation and management, is presented. The patient’s treatment course consisted of three craniotomies, 3,600-cGy fractionated radiation and two courses of glucocorticoid therapy. In addition to disease surveillance using clinical examination and imaging, this case represents the first description of the clinical utility of analyzing changes in an inflammatory blood marker, the erythrocyte sedimentation rate, which coincided with recurrence and response to therapy.

## Introduction

Plasma cell granuloma is a rare entity describing a non-neoplastic lesion featuring histological proliferation of polyclonal plasma cells, lymphocytes, neutrophils, eosinophils and histiocytes in a fibrotic background (1). While this mass lesion is reported to occur in nearly every organ in the body, plasma cell granulomas most frequently present in the lungs. The underlying cause and natural history of these lesions remains unknown and treatment options include excision, radiation and steroids (2). The current case report presents the longest known follow-up period in the literature for an intracranial plasma cell granuloma. In addition, the course of the patient’s disease following multimodal therapy, including surgery, radiation and steroids, is detailed. The study was approved by the Wake Forest University School of Medicine Institutional Review Board (Winston-Salem, NC, USA). Written informed consent was obtained from the patient’s family.

## Case report

### Patient characteristics

A 55-year-old Caucasian female with a history of hypertension, chronic otitis and mastoiditis presented to an outside institution with a three-week history of bitemporal frontal headaches and left-sided hearing loss. On examination, the patient was awake and alert but was noted to have difficulty with speech. Bilateral papilledema was identified by fundoscopy. The remainder of the exam was nonfocal and without further neurological deficits. A computed tomography with contrast revealed a 5×5-cm heterogeneously enhancing mass in the left temporal lobe with a large amount of associated left hemisphere edema and left-to-right shift. The patient underwent surgery for further management.

### Pathological analysis

Pathological analysis of a subtotal resection demonstrated perivascular and intraparenchymal collections of lymphocytes and plasma cells set within a densely fibrotic background. Neurons and reactive astrocytes were entrapped within the inflammatory lesion. The lymphocytes and plasma cells appeared small and mature with no histological evidence of lymphoma or myeloma. There was no evidence of vasculitis or amyloid deposits on the routine stains. Immunohistochemical studies revealed the presence of κ (marginally predominant) and λ light chains, confirming the polyclonal nature of the plasma cell infiltrate, along with a mixed population of T and B cells, consistent with an inflammatory process. Stains for microorganisms and Epstein-Barr virus were negative.

Further work-up included a bone marrow biopsy, serum electrophoreses (negative for monoclonal and polyclonal gammopathy) and β-2 microglobulin, which were all within normal limits. A final diagnosis of intracranial plasma cell granuloma was made.

### Clinical course

The patient’s course was punctuated by several recurrences over a 14-year follow-up period. The first recurrence in the left temporal region was detected by surveillance magnetic resonance imaging (MRI) at three years and treated with another surgical resection and 3,600-cGy adjuvant radiation therapy fractionated over four weeks. A second recurrence was again detected by surveillance MRI at seven years and treated with a third resection of a 3×5-cm mass. Pathology was again consistent with recurrent plasma cell granuloma. One month later, the patient presented to an ophthalmologist with decreasing vision in the left eye. A small blind spot progressed to complete loss of vision out of the left eye over the next eight months. Two months later, the patient noted decreasing vision in the right eye and was referred to Wake Forest University School of Medicine for further management.

**Figure 1:**
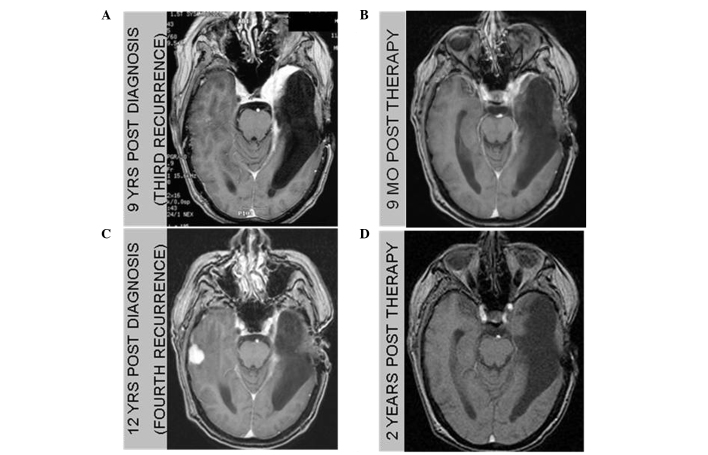
Axial MRI T1 with contrast images. (A) Third recurrence, nine years following diagnosis, of an enhancing lesion in the anterior temporal fossa involving the left optic nerve. (B) Resolution of the lesion following 9 months of steroid therapy. (C) Fourth recurrence, 12 years following diagnosis, consisting of a new contrast-enhancing lesion in the contralateral temporal lobe. (D) Two-year follow-up showing complete sustained resolution of the lesion following seven months of steroid therapy.

Now at nine-years post-diagnosis, work-up at our facility, including MRI imaging and erythrocyte sedimentation rate (ESR) measurement (Fig. 1A), revealed a third recurrence constituted by a new area of enhancement, somewhat anterior to the resection cavity in the anterior temporal fossa, surrounding the orbital apex and involving the left optic nerve along with an elevated ESR. The patient refused additional surgical intervention and was not considered to be a candidate for additional radiation therapy. The treatment plan consisted of 4 mg dexamethasone four times a day, which was steadily reduced over two months due to a steroid-induced myopathy and psychosis. Despite persistent visual loss, a six-month post-steroid MRI study demonstrated a reduction in size of the lesion, constituting a partial response, accompanied by a decrease in the ESR from an initial value of 56 to 10 mm/h. This apparent response prompted treatment with oral prednisone at a dose of 10 mg every other day that was well tolerated and without side effects. A repeat MRI three months later revealed resolution of the mass (Fig. 1B). The sedimentation rate remained low at 11 mm/h. Steroids were tapered over the subsequent two months.

Twelve-years following the initial diagnosis and 1.5 years after steroid treatment, a surveillance MRI demonstrated a new contralateral peripherally enhancing mass surrounded by vasogenic edema in the right temporal lobe (Fig. 1C). Of note, surveillance imaging noted opacification of the right mastoid air cells six months prior to the occurrence of this lesion representing a potential infectious etiology seeding eventual granuloma formation. This fourth recurrence was treated with 80 mg oral prednisone daily. Following two months of therapy, the patient had a partial response with a decline in the size of the lesion by 50%. Prednisone was tapered and eventually discontinued over the next five months. The ESR was 42 mm/h during this recurrence and remained elevated.

**Figure 2:**
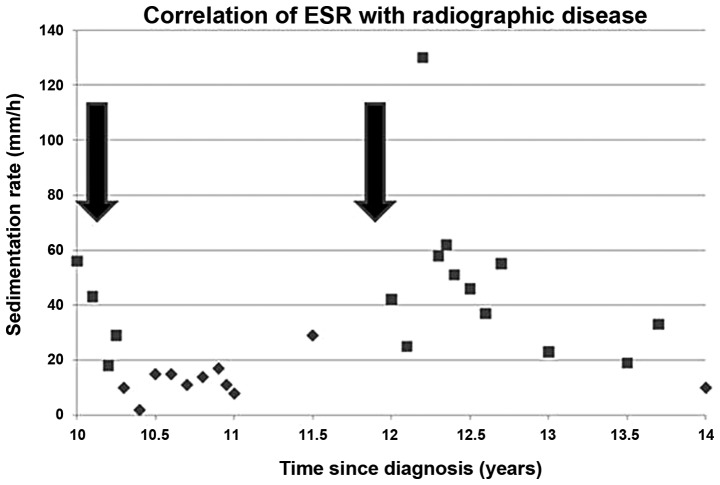
Timeline depicting periods of radiological evidence of active disease (large squares) and resolved disease (small diamonds) associated with ESR. Recurrences are denoted by large arrows. Periods of active disease were associated with an increased ESR. ESR, erythrocyte sedimentation rate.

Fourteen years following the initial diagnosis and two years following the patient’s latest course of prednisone, a repeat brain MRI revealed a near complete response (Fig. 1D). The sedimentation rate was 10 mm/h (Fig. 2). Several months later, at 69 years-old, the patient unexpectedly succumbed to a probable myocardial infarction, arrhythmia or pulmonary embolus at home, following complaint of substernal chest pain. The patient did not undergo an autopsy.

## Discussion

The number of reported intracranial cases of plasma cell granuloma reviewed in the literature is ~50 (2–5). The paucity of long-term follow-up cases may result in an incomplete understanding of the natural history and recurrence management of this rare disease. In the present study, the patient was followed until death, fourteen years following the initial diagnosis. Furthermore, the individual suffered four disease recurrences, adding to the eight reported cases of relapsing intra-axial plasma cell granuloma (3). Treatment of the recurrences consisted of two craniotomies, 3,600-cGy fractionated radiation and two courses of glucocorticoid therapy.

Histopathological analysis remains the gold standard for achieving the diagnosis of plasma cell granuloma. Tissue sections in the present patient contained a perivascular infiltrate of lymphocytes and polyclonal plasma cells, which is characteristic of plasma cell granulomas (3,6,7). A slight predominance of κ as compared to λ light chains was noted, and this has also been found in other cases of plasma cell granuloma. Given the inflammatory nature of these lesions, the diagnostic work-up includes a search for microorganisms or a systemic inflammatory condition. The current patient’s history of chronic mastoiditis and otitis on the same side of the lesion raises suspicion of the possibility of undetected organisms.

Monitoring for response to therapy and recurrences traditionally relies on clinical exam and surveillance imaging. To the best of our knowledge, this case represents the first time ESR has been reported to rise and fall in concert with disease recurrence, presence and resolution. Rising ESR values appeared to be specific to the disease and were not the result of surgical intervention, as these recurrences were treated with steroids. Further, the decline in the ESR appeared to be specific to disease activity rather than the result of a systemic immune suppression, as the values correlated with imaging changes. The literature reports elevation of inflammatory markers, ESR and CRP, in patients with plasma cell granulomas; however, the marker levels were analyzed at diagnosis and not serially followed, with only one case series describing a decrease in ESR following surgical management of pulmonary plasma cell granulomas (8–10). Trending ESR may complement imaging with regard to disease activity, treatment response or impending radiographical recurrence.

This unique case of a plasma cell granuloma was treated for four recurrences over the course of 14 years. Each treatment resulted in a complete, lengthy, but unsustained response. Relapses and responses of the disease were detected by imaging and ESR, which may prove supplemental to imaging studies for evaluating disease activity.
